# Supplementary material for: Pathogenesis, Clinical Features, and Treatment of Patients with Myelin Oligodendrocyte Glycoprotein (MOG) Autoantibody-Associated Disorders Focusing on Optic Neuritis with Consideration of Autoantibody-Binding Sites: A Review
Source: Int J Mol Sci. 2023 Aug 29;24(17):13368. doi: 10.3390/ijms241713368 (PMC10488293; doi:10.3390/ijms241713368)
Supplement: Supplementary file 1 [file ijms-24-13368-s001.zip › ijms-2422383-supplementary.pdf]

Supplementary Table S1. Comparison of Anti-MOG and Anti-AQP4 Antibody Ratios in Optic Neuritis Patients Across Different Regions. [Ref.15, 17-19]

Optic Neuritis Patients	MOG-Ab (+)	AQP4-Ab (+)
US (Mayo Clinic) (n=246)	32 (13)	47 (19)
Korea (n=270)	17 (6.3)	49 (18.1)
Germany (n=522)	87 (16.7)	83 (15.9)
Japan (n=531)	53 (10)	64 (12)

A cross-sectional cohort study conducted in different populations across the world showed that the prevalence rates were similar across all populations studied..

Supplementary Figure S1. Age distribution in MOG-positive/AQP4-positive/double negative group.

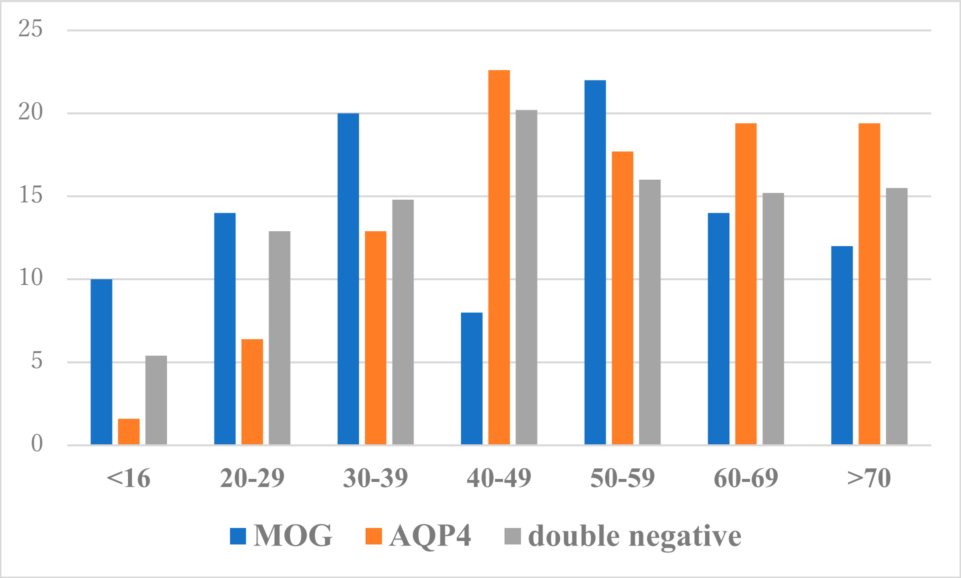

The anti-AQP4 autoantibody positivity increased with age whereas the anti-MOG autoantibody positivity exhibited biphasic peaks in the fourth and sixth decades of life.
